# Surface Modification of Feldspathic Ceramic Used for Minimally Invasive Restorations: Effect of Airborne Particle Type on the Surface Properties and Biaxial Flexural Strength

**DOI:** 10.3390/ma17153777

**Published:** 2024-08-01

**Authors:** Moritz Hoffmann, Felix Schmeiser, Mustafa Borga Donmez, John Meinen, Bogna Stawarczyk

**Affiliations:** 1 Department of Prosthetic Dentistry, Dental School, Ludwig-Maximilians-Universität München, 80336 Munich, Germany; moritz.hoffmann@med.uni-muenchen.de (M.H.); felix.schmeiser@med.uni-muenchen.de (F.S.); john.meinen@med.uni-muenchen.de (J.M.); bogna.stawarczyk@med.uni-muenchen.de (B.S.); 2 Department of Reconstructive Dentistry and Gerodontology, School of Dental Medicine, University of Bern, 3010 Bern, Switzerland; 3 Department of Prosthodontics, Faculty of Dentistry, Biruni University, 34015 Istanbul, Turkey

**Keywords:** airborne particle abrasion, felspathic ceramic, flexural strength, roughness, surface free energy

## Abstract

This study aimed to evaluate the effect of airborne particle abrasion with different particles on the surface free energy, roughness, and biaxial flexural strength of a feldspathic ceramic by comparing it with hydrofluoric acid etching, the standard surface treatment, and polishing. Square-shaped feldspathic ceramic specimens (12 mm × 12 mm × 1.2 mm) were divided into subgroups as airborne particles abraded with alumina (AO3a, AO3b, AO25, AO50a, AO50b, AO90, AO110a, AO110b, AO120a, and AO120b), silica (SO50a, SO50b, SO100, and SO100/200), or nutshell granule (NS100/200), hydrofluoric acid etched, and polished (n = 12). Surface free energy (n = 5), roughness (n = 5), biaxial flexural strength (n = 12), and Weibull moduli (n = 12) were investigated. Data were evaluated with 1-way ANOVA and Tukey HSD tests, and possible correlations were investigated with Pearson’s correlation (α = 0.05). SO100/200 mostly had lower surface free energy (*p* ≤ 0.011), and polishing and etching led to higher surface free energy than AO3a, AO3b, and AO120a (*p* ≤ 0.031). Polished, SO100, and SO50b specimens mostly had lower roughness and AO125 had the highest roughness (*p* ≤ 0.029). SO100/200 mostly had lower biaxial flexural strength (*p* ≤ 0.041), and etched specimens had higher biaxial flexural strength than AO120a, AO120b, and SO50b (*p* ≤ 0.043). AO3b had the highest (33.56) and AO120b had the lowest (11.8) Weibull modulus. There was a weak positive correlation between the surface free energy and the biaxial flexural strength (r = 0.267, *p* = 0.011). A larger particle size mostly resulted in higher roughness, which was also affected by the particle shape. Most of the test groups had similar biaxial flexural strength to that of the hydrofluoric acid-etched group. Therefore, for tested feldspathic ceramic, airborne particle abrasion with tested parameters may be a suitable alternative without causing any further damage.

## 1. Introduction

Esthetic demands of patients and clinicians have increased the popularity of computer-aided design and computer-aided manufacturing (CAD-CAM) technologies, which facilitate indirect rehabilitation of a restoration in a single appointment [1,2]. This tendency towards esthetic restorations has also increased the application of ceramics [3]. Among those, feldspathic ceramics are frequently preferred for minimally invasive and esthetic restorations such as inlays, onlays, and laminate veneers [4]. These ceramics are mainly composed of a crystalline phase and a vitreous matrix [5].

Feldspathic ceramics should be cemented adhesively due to their lower flexural strength [6], particularly when compared with other glass ceramics [7], as adhesive cementation has been reported to improve the mechanical properties of ceramics [6]. A clinically optimal bond strength between the restorative material and the resin composite cement is critical for the success and sustainability of indirect restorations [8,9]. High bond strength ensures the marginal adaptation, sealing, retention, and fracture resistance of indirect restorations [5,10], which is particularly important considering the brittle nature of feldspathic ceramics [3]. Different surface treatment methods, such as airborne particle abrasion, hydrofluoric acid etching, single-step ceramic primer, and laser application, have been proposed to increase the bond strength between the restorative material and the resin composite cement [10,11,12]. Hydrofluoric acid etching has been considered the standard surface treatment for silicate-based ceramics [13,14]; however, possible hazards of hydrofluoric acid, such as burns to the skin due to hydrogen ion degeneration and corrosion, have been reported [15]. These hazardous effects might be more critical when hydrofluoric acid is used for intraoral repairs. In addition, hydrofluoric acid contamination of dentin and enamel during intraoral repairs might lower the bond strength [16,17].

Airborne particle abrasion may be a suitable alternative to hydrofluoric acid etching, as this method also increases the surface area of ceramics for higher bond strength and can also be used intraorally [18,19]. However, the parameters of airborne particle abrasion, such as the amount of air pressure, duration of the process, and angle of the nozzle, may vary [4], and there are particles of different sizes that can be used [20,21]. Previous studies on airborne particle abrasion of feldspathic ceramics have investigated the effect of different parameters of the process on the surface topography and bond strength [4], color stability [21], and mechanical properties [12,22] of feldspathic ceramics. However, knowledge on the effect of particle size and shape on different properties of CAD-CAM feldspathic ceramics is lacking. In addition, increased particle size might deteriorate the mechanical properties of ceramics due to increasing microcracks. Therefore, the aim of the present study was to evaluate how the surface and mechanical properties of a CAD-CAM feldspathic ceramic when airborne particles were abraded with particles in different shapes and sizes were affected by comparing them with hydrofluoric acid-etched and polished ones. The null hypotheses were that the surface treatments would not affect the (i) surface free energy, (ii) surface roughness, or (iii) biaxial flexural strength of tested feldspathic ceramic.

## 2. Materials and Methods

### 2.1. Specimen Preparation

**Figure 1:**
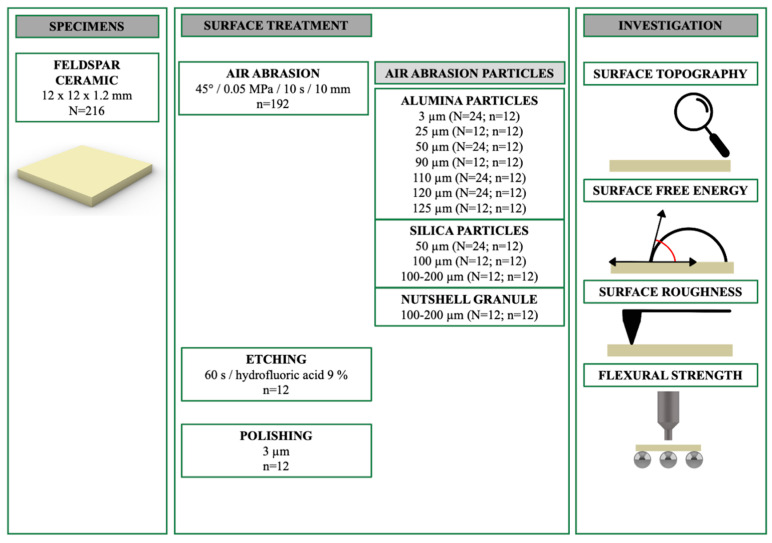
Overview of the study design.

Figure 1 presents the overview of this investigation. Feldspathic ceramic blocks (VITABLOCS Mark II, VITA Zahnfabrik, Bad Säckingen, Germany) were wet-sliced by using a precision cutter (Secotom-50, Struers, Ballerup, Denmark) to obtain 216 square-shaped specimens (12 mm × 12 mm × 1.2 mm). A recent study has evaluated this material and reported the chemical composition as SiO_2_: 56–64 wt%, Al_2_O_3_: 20–23 wt%, Na_2_O: 6–9 wt%, K_2_O: 6–8 wt%, and CaO: 0.3–0.6 wt%. The same study also reported that the mean fracture toughness, Martens hardness, and indentation modulus of this material were 1.25 MPa√m, 3640 MPa, and 59.5 GPa [23]. One surface of each specimen was ground down under water cooling by using grinding sheets (up to SiC Papers #500, Struers, Ballerup, Denmark) and a grinding machine (Abramin, Struers, Ballerup, Denmark). The other side of each specimen was polished with polishing pads (MD Largo, Struers, Ballerup, Denmark) and diamond suspensions (DiaPro Largo 3 µm and 9 µm, Struers, Ballerup, Denmark). Thereafter, each specimen was ultrasonically cleaned in alcohol (Isopropanol 96% *v*/*v*, Otto Fischar, Saarbrücken, Germany) and divided into 18 subgroups according to the surface treatments as airborne particles abraded by using 16 different particles, polished, and hydrofluoric acid-etched (n = 12).

Table 1 lists detailed information on the particles tested in the present study, which are evaluated for their size and shape by using a digital microscope (VHX-970F, Keyence, Osaka, Japan) under ×200 magnification. All airborne particle abrasion procedures were performed from a distance of 10 mm for 10 s with an angle of 45° and 0.05 MPa pressure (Basic quattro IS, Renfert GmbH, Hilzingen, Germany). All air-abraded specimens were then ultrasonically cleaned for 3 min (Transistor/Ultrasonic T-14, L&R Manufacturing, Rengsdorf, Germany) in distilled water and dried with a cellulose wipe to ensure that the surfaces were free of dust and particles before measurement. The specimens of the hydrofluoric acid etching group were treated with a hydrofluoric acid gel (9% Ultradent Porcelain Etch, Ultradent Products Inc., South Jordan, UT, USA) for 60 s. The gel was removed under running water, and the specimens were ultrasonically cleaned (Sonorex Digitec DC, Bandelin, Berlin, Germany) in distilled water for 3 min. The unpolished surface of the specimens in the polished group was also polished, as mentioned above, and then ultrasonically cleaned in distilled water. The same digital microscope was used to evaluate the surface topography of one additional specimen from each group under ×200 magnification.

### 2.2. Surface Free Energy

The sessile drop technique was used to evaluate the surface free energy of 5 specimens from each group at room temperature by using a drop shape analysis system (DSA 25 EasyDrop, Krüss, Hamburg, Germany) with 3 liquids of different polarity: deionized water, 99% diiodomethane (Sigma-Aldrich Chemie GmbH, St. Gallen, Switzerland), and ethylene glycole (AppliChem GmbH, Darmstadt, Germany). The contact angle measurement was performed 5 s after the drop application, and each specimen’s measurement was performed 3 times with each liquid. Either tangent-1 (deionized water) or circular (diiodomethane and ethylene glycole) was used for contact angle calculation. The surface free energy was calculated using the Ström database and the Owens–Wendt–Rabel–Kaelble method [24].

### 2.3. Surface Roughness

A contact profilometer (MarSurf M400, Mahr GmbH, Göttingen, Germany) was used to analyze the roughness values of the same 5 specimens from each group that were used to evaluate surface free energy. Six measurements (3 horizontal and 3 vertical) with a track length of 4 mm and a tip measuring force of 0.75 mN were performed for each specimen [25]. The arithmetic mean of these 6 measurements was recorded as the roughness value of each specimen.

### 2.4. Biaxial Flexural Strength (σ)

A universal testing machine (Zwick 1445, Zwick-Roell, Ulm, Germany) and its proprietary software (testXpert II V. 3.6, Zwick-Roell, Ulm, Germany) were used to evaluate the biaxial flexural strength in a piston-on-three ball method. The steel balls had a diameter of 3.2 mm and were arranged at an angle of 120° to each other on a circle 10 mm in diameter. The force was applied with a stainless-steel piston that had a diameter of 1.6 mm, and the load was applied at 1 mm/min until the specimen fractured. The load was applied to the unmodified surface of each specimen as tensile stresses occurred on the bottom surface of the specimen during the test. To calculate the biaxial flexural strength, the formula was adapted according to the specimen geometry, and the pre-factor f was calculated for the dimensions of the specimens (12 mm × 12 mm × 1.2 mm) [26].

$$f\;\left(\tau ,\;\nu \right)=0.323308+\frac{\left(1.30843+1.44301\;\cdot \;\nu \right)\;\cdot \;\left(1.78428-3.15347\;\cdot \;\tau +6.67919\;\cdot \;\tau^{2}-4.62603\;\cdot \;\tau^{3}\right)}{1+1.71955\;\cdot \;\tau }$$

$$\sigma max=f(\tau ,\;\nu )\;\cdot \;\frac{F\;}{t^{2}}$$

σmax is the biaxial flexural strength in MPa, F is the force at the moment of fracture of the specimen in N, t is the specimen thickness in mm, f is the f-function, ν is the Poisson’s ratio (0.18) [27], and τ is the radius of the support disk in mm. The number of fragments was categorized according to their relative frequency, and they were analyzed by using a digital microscope (VHX-970F, Keyence AG, Osaka, Japan).

### 2.5. Statistical Analysis

The distribution of data was analyzed using Kolmogorov–Smirnov test. Considering that all data had a normal distribution, further analyses were performed using 1-way ANOVA and Bonferroni-corrected post hoc Tukey HSD tests. Any possible correlation between the investigated outcomes was analyzed with Pearson’s correlation [28]. All analyses were performed using statistical analysis software (SPSS v29, IBM Corp., Seattle, WA, USA) with a significance level of α = 0.05. Weibull moduli were calculated using the least squares method [29]. The relative frequency of fragments together with the corresponding 95% confidence intervals were analyzed using the Ciba-Geigy table.

## 3. Results

**Figure 2:**
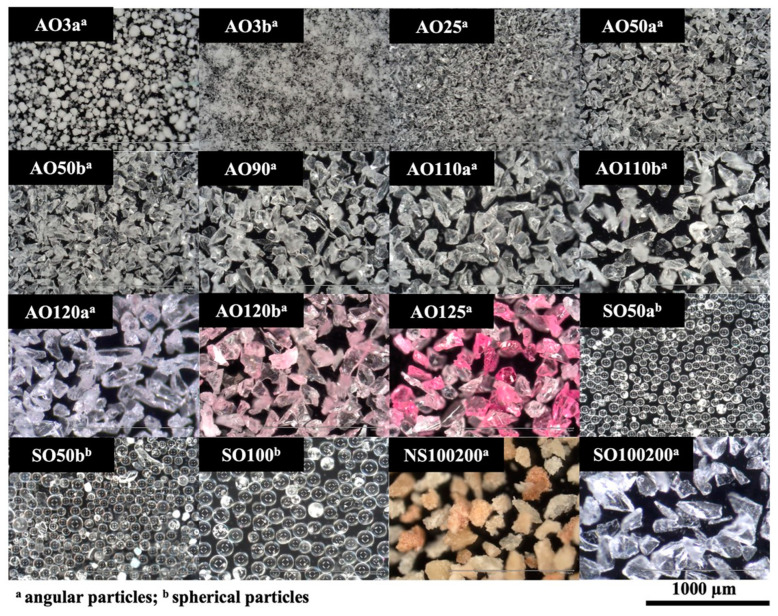
Digital microscope images of particles used for the airborne particle abrasion process (images taken under 200× magnification).

Under microscopic evaluation, tested abrasive particles showed different geometries, even if they were made of the same material or had the same particle size and shape. AO3a formed agglomerates of different sizes, whereas AO3b retained its powder-like structure based on the individually present particles. AO25 particles had a somewhat similar shape to that of AO3b particles, with an evident increase in particle size. The particles started to become more perceptible when the size was 50 µm or higher. However, there was a major difference between AO and SO particles of the same size, as AO particles had geometrical shapes with edges, whereas SO particles were spherical. Other than their size, the particles of AO120a, AO120b, and AO125 had distinct colors that separated them from the other AO particles. Even though single-sized SO particles were spherical, the particles of SO100 were visibly larger than those of SO50a and SO50b. In addition, geometrical deviations from this spherical shape were more evident with SO50a. The particles of SO100/200 and NS100/200 had a similar shape to that of the AO particles. However, the NS100/200 particles also had a different color and, additionally, a higher range of particles of different sizes within an air abrasion powder (Figure 2).

**Figure 3:**
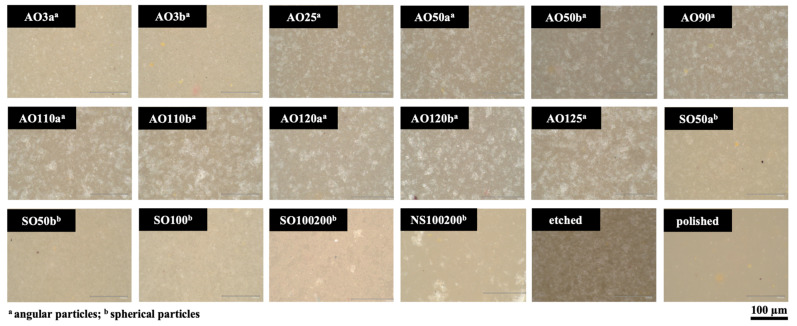
Digital microscope images of the surface topography of one specimen from each test group (images taken under 200× magnification).

When the surface of one specimen from each test group was evaluated, it was observed that the polished specimen had the smoothest surface. A somewhat similar surface topography was visible for AO3a, AO3b, SO50a, SO50b, SO100, and SO100/200, and NS100/200 had a slightly rougher surface appearance. The surfaces of these specimens had a higher number of brighter areas than the polished specimen. The increased particle size in groups abraded with AO led also increased the number and size of the bright areas, which was similar to that of the hydrofluoric acid-etched specimen (Figure 3).

Significant differences were observed among test groups for each tested outcome (*p* < 0.001). When surface free energy was considered, SO100/200 had lower values than all groups (*p* ≤ 0.011), except for AO3a, AO3b, AO50a, AO120a, and SO50a (*p* ≥ 0.142). AO120a and AO3b had lower values than SO50b, NS100/200, polished, and hydrofluoric acid-etched specimens, while AO3a had lower values than polished and hydrofluoric acid-etched specimens (*p* ≤ 0.031). Every other pairwise comparison was nonsignificant (*p* ≥ 0.072) (Table 2).

Polished, SO100, and SO50b specimens had lower roughness than all specimens (*p* ≤ 0.029), other than those in groups AO3b, SO50a, and NS100/200 (*p* ≥ 0.290), which also had similar values to AO3a (*p* ≥ 0.097). SO100/200, hydrofluoric acid-etched, and AO25 specimens had higher roughness than polished SO100, SO50b, AO3a, AO3b, SO50a, and NS100/200 and had lower roughness than the remaining groups (*p* < 0.001). AO120a had higher roughness than AO50b (*p* = 0.019), AO110a had higher roughness than AO50b, AO90, and AO120b (*p* ≤ 0.038), and AO110b had higher roughness than AO50b, AO50a, AO90, and AO120b (*p* ≤ 0.033). AO125 had the highest roughness among the tested materials (*p* < 0.001). Every other pairwise comparison was non-significant (*p* ≥ 0.084) (Table 2).

**Figure 4:**
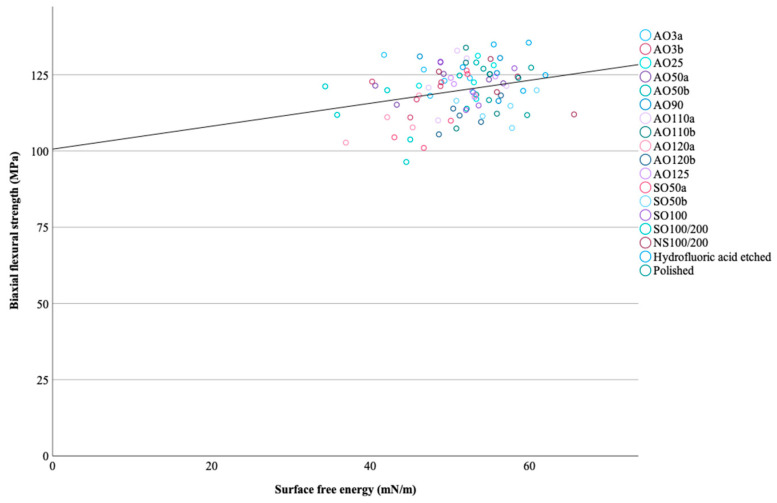
Graphical representation of the correlation between biaxial flexural strength and surface free energy.

**Figure 5:**
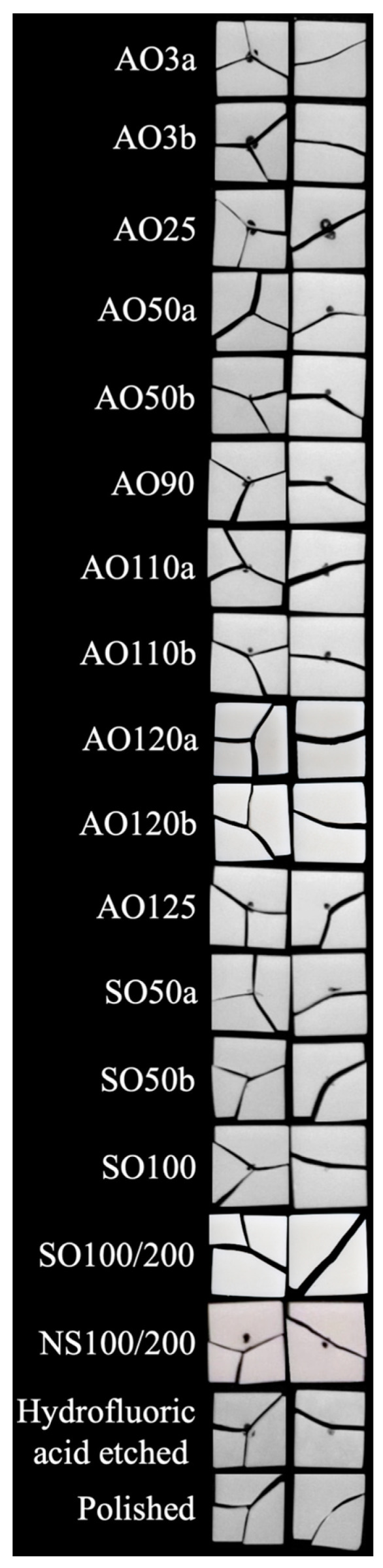
Representative image of one specimen from each group with a different number of fragments.

SO100/200 had lower biaxial flexural strength than all groups (*p* ≤ 0.041) other than AO120a, AO120b, and SO50b (*p* ≥ 0.129). AO120b had lower biaxial flexural strength than AO50a, AO110a, and hydrofluoric acid-etched specimens (*p* ≤ 0.035), while AO120a and SO50b also had lower biaxial flexural strength than hydrofluoric acid-etched specimens (*p* ≤ 0.043). Every other pairwise comparison was nonsignificant (*p* ≥ 0.054). The Weibull moduli of test groups ranged between 33.56 (AO3b) and 11.77 (AO120b) (Table 2). Pearson’s correlation analyses only revealed a weak positive correlation was observed between the surface free energy and the biaxial flexural strength of tested specimens (r = 0.267, *p* = 0.011) (Figure 4). Figure 5 shows fragments of fractured specimens for each surface treatment subgroup. Two-piece fragments of the fractured specimens were mostly more frequent than three-piece fragments (Table 2).

## 4. Discussion

The aim of the present study was to evaluate whether airborne particle abrasion with particles of different sizes and geometries can be a suitable surface treatment alternative to hydrofluoric acid etching from a mechanical standpoint. Therefore, the surface properties and biaxial flexural strength of a CAD-CAM feldspathic ceramic when subjected to different surface treatments that include airborne particle abrasion with particles of different material types, sizes, and geometries, hydrofluoric acid etching, and polishing were investigated. Significant differences were observed among the specimens treated with tested protocols within each investigated outcome. Therefore, the null hypotheses were rejected.

Surface free energy is a critical parameter to be considered for the flow of the adhesive used and adequate bond strength, as the surface tension of the adhesive cannot be changed. Thus, changing the surface free energy of the substrate surface may improve the bonding between the resin cement and the restorative material [9]. In the present study, airborne particle abrasion with SO100/200 mostly led to lower surface free energy values; therefore, it can be hypothesized that when tested feldspathic ceramic is abraded with this particle, it would also have lower bond strength than when treated with most of the other surface treatments tested in this study. The specimens treated with AO had similar surface free energy regardless of the particle size; thus, it can be stated that the effect of the particle size of AO did not affect the surface free energy of tested the feldspathic ceramic. However, when SO particles were considered, the specimens abraded with SO100/200 had significantly lower surface free energy than SO50b and SO100 and non-significantly lower surface free energy than SO50a. These results may be interpreted as 100 µm being the threshold for SO particle size while abrading tested feldspathic ceramic and spherical shape being more favorable to achieving higher surface free energy. However, these speculations need to be supported by studies that involve larger SO particles without the presence of smaller particles in the mixture and angular SO particles with smaller particle sizes like 50 µm and 100 µm. When particles of the same size (50 µm) were considered, AO and SO particles led to similar surface free energy, and particles of the same size led to similar surface free energy within AO and SO particles. NS100/200 only led to statistically higher surface free energy than AO3b, AO120a, and SO100/200; however, it may be considered as an alternative to tested abrasive particles that is ecological and less harmful (silicosis), given that none of the abraded specimens had a higher mean surface free energy than those abraded with NS100/200.

AO125 resulted in the highest roughness among tested surface treatments and considering that a positive correlation between roughness and shear bond strength of CAD-CAM ceramics has been reported previously [13], airborne particle abrasion with AO125 may be the most suitable treatment for intraoral repairs of tested feldspathic ceramic. There was no clear trend on how particle size affected the roughness of specimens treated with AO particles; however, particles bigger than 50 µm had higher roughness than AO3a, AO3b, and AO25. When SO particles were considered, the roughness values were similar among groups with a single particle size. However, SO100/200 resulted in higher roughness values than those groups. This result may be related to the difference in the shape of the particles, as only SO100/200 had edges and the specimens treated with AO particles, which also had edges, had either significantly or non-significantly higher roughness than SO50a, SO50b, and SO100. When particles with mixed particle sizes were considered, SO100/200 resulted in higher roughness than NS100/200. In fact, NS100/200 led to lower roughness than most of the surface treatments tested in the present study and therefore might not be an ideal treatment to roughen a restoration’s inner surface when compared with most of the tested surface treatments.

SO100/200 only had similar biaxial flexural strength to the AO120a, AO120b, and SO50b and had lower biaxial flexural strength than the remaining groups, while hydrofluoric acid-etched specimens had higher biaxial flexural strength than the AO120a, AO120b, and SO50b groups. However, the maximum meaningful mean difference among test groups was 17 MPa. When particles of different sizes were considered, the maximum meaningful mean difference was 11 MPa for AO (AO110a and AO120b) and was 15.09 MPa for SO (SO100 and SO100/200) particles. The effect of particle type did not affect the biaxial flexural strength of the tested feldspathic ceramic, as AO50a, AO50b, SO50a, and SO50b had similar values. Considering that all specimens had mean biaxial flexural strength values that were higher than 100 MPa, which was referred to as the threshold value for a ceramic to be used for a monolithic single-unit anterior or posterior prosthesis to be adhesively cemented according to the International Organization for Standardization standard 6872:2015 [30], and the fact that meaningful mean differences among tested materials were relatively low, it can be speculated that these differences may not have clinical relevance. In addition, two-piece fragments were more frequent for most of the subgroups, suggesting that tested surface treatments may not impair the mechanical properties of tested feldspathic ceramic with microcracks that might propagate and lead to failure. However, there was evident variability in measured Weibull moduli, which represents the reliability of the test groups. Therefore, this hypothesis should be supported by studies on the long-term fatigue behavior of tested feldspathic ceramic when tested surface treatments and different resin cements were used, considering that resin cement would seal microcracks and increase the mechanical properties of the ceramic by filling the surface irregularities.

To the authors’ knowledge, the present study was the first on how airborne particle abrasion powders in different material types, sizes, and shapes affect different properties of a CAD-CAM feldspathic ceramic, which complicates comparisons with previous studies. However, there are studies on the mechanical properties of feldspathic ceramics used for laminate veneers when airborne particles are abraded with particles of different sizes [12,22]. One of those studies reported that particles with a 50-µm diameter had improved reliability in terms of fracture strength [12], while the other study mentioned that 50-µm diameter particles were favorable for improving surface roughness without compromising flexural strength [22]. In a previous study on the effect of airborne particle abrasion on the shear bond strength of composites to a feldspathic ceramic, it was concluded that the pressure of the abrasion process did not affect the shear bond strength when 50 µm diameter alumina was used [4]. A recent study evaluated how particles of different sizes and compositions affected the color stability of different CAD-CAM ceramics, including the one tested in the present study [21]. The authors [21] reported that tested particles did not affect the color stability of the feldspathic ceramic, whereas that of a resin nanoceramic and a flexible hybrid ceramic was affected.

a priori power analysis to determine the number of specimens could not be performed, given that the present study was the first on the effect of airborne particle abrasion particles on different properties of a CAD-CAM feldspathic ceramic. Even though this is a limitation of the present study, significant differences were observed among test groups within each outcome, and therefore, the authors think that the number of specimens in each group is justified. It should also be mentioned that other parameters of the abrasion process were standardized, and previous studies have shown the effect of those parameters on the mechanical properties of feldspathic ceramics [12,22]. Another limitation was that only one type of CAD-CAM ceramic was tested, and different materials may lead to different results. In the present study, square-shaped specimens were fabricated by wet-slicing CAD-CAM blocks. However, in actual clinical situations, milling units with different numbers of axes are used to fabricate restorations with more complex geometries, and the specimen fabrication method and geometry may affect the investigated outcomes [31]. All specimens were polished in the present study, and glazing may affect the results. In addition, no aging was performed, and how tested properties are affected in the long term is unknown. Finally, the present study only focused on how airborne particle abrasion with different particles affected the surface properties and biaxial flexural strength. Future studies should investigate the effect of airborne particle abrasion with tested particles and different parameters on other mechanical properties, optical properties, and repair bond strength of CAD-CAM ceramics with different chemical compositions to elaborate on the findings of the present study. In addition, future studies should include scanning electron microscope images of surfaces treated with tested methods before and after fracture to substantiate the hypothesis that airborne particle abrasion does not affect the formation of surficial microcracks in tested feldspathic ceramic.

## 5. Conclusions

The surface roughness of tested feldspathic ceramics mostly increased when particles with larger sizes or angular shapes were used. Silica particles with mixed particle sizes (SO100/200) should not be preferred to abrade tested feldspathic ceramic, given the lower surface free energy and biaxial flexural strength values. Airborne particle abrasion with tested parameters and particles except for SO100/200 may be suitable to abrade tested feldspathic ceramic as it leads to similar biaxial flexural strength to that of the hydrofluoric acid-etched group.

## Figures and Tables

**Table 1:** List of particles used in this study.

	Particle Size	Particle Shape	Abbreviation	Manufacturer	LOT-No.
**Alumina particles**					
Nabalox 205-71	3 µm	Angular-Edged	AO3a	Nabaltec, Schwanndorf, Germany	11089
Nabalox 230	3 µm	Angular-Edged	AO3b		09764
Cobra	25 µm	Angular-Edged	AO25	Renfert, Hilzingen, Germany	2414914
Strahlkorund	50 µm	Angular-Edged	AO50a	Orbis, Münster, Germany	21.545337
Cobra	50 µm	Angular-Edged	AO50b	Renfert, Hilzingen, Germany	2430355
Cobra	90 µm	Angular-Edged	AO90		2430353
Cobra	110 µm	Angular-Edged	AO110a		2433408
Korox	110 µm	Angular-Edged	AO110b	Bego, Bremen, Germany	2484713
Korund rosa	120 µm	Angular-Edged	AO120a	Renfert, Hilzingen, Germany	Experimental
Korund strong rosa	120 µm	Angular-Edged	AO120b		Experimental
Cobra	125 µm	Angular-Edged	AO125		2414912
**Silica particles**					
Perlablast	50 µm	Spherical	SO50a	Bego, Bremen, Germany	L08879
Rolloblast	50 µm	Spherical	SO50b	Renfert, Hilzingen, Germany	L09376
Rolloblast	100 µm	Spherical	SO100		L08098
Glass granule	100–200 µm	Angular-Edged	SO100/200		Experimental
**Nutshell granule**					
Nutshell granule	100–200 µm	Angular-Edged	NS100/200	Renfert, Hilzingen, Germany	Experimental

**Table 2:** Mean ± standard deviation (95% confidence intervals) surface free energy (mN/m), roughness (µm), biaxial flexural strength (MPa) values, Weibull moduli, and relative frequency of fragments within test groups.

Test Groups	Surface Free Energy (mN/m)	Surface Roughness (µm)	Biaxial Flexural Strength (MPa)	Weibull Modulus	Three-Piece Fragments (n/%) [95% Confidence Intervals]	Two-Piece Fragments (n/%) [95% Confidence Intervals]
AO3a	47.5 ± 4.0 ^abc^ (42.6–52.5)	0.28 ± 0.06 ^b^ (0.21–0.36)	120 ± 5 ^bcd^ (113–127)	16.3	6/50% [20–80%]	6/50% [20–80%]
AO3b	45.7 ± 3.6 ^ab^ (41.3–50.2)	0.16 ± 0.03 ^ab^ (0.12–0.20)	121 ± 5 ^bcd^ (113–128)	33.6	6/50% [20–80%]	6/50% [20–80%]
AO25	52.3 ± 4.0 ^bcd^ (47.8–56.7)	0.58 ± 0.14 ^c^ (0.41–0.75)	121 ± 6 ^bcd^ (113–129)	19.0	4/33% [8–67%]	8/67% [33–92%]
AO50a	49.0 ± 7.0 ^abcd^ (40.2–57.7)	1.00 ± 0.07 ^def^ (0.91–1.08)	123 ± 4 ^cd^ (116–128)	15.1	4/33% [8–67%]	8/67% [33–92%]
AO50b	53.0 ± 1.4 ^bcd^ (51.2–54.8)	0.85 ± 0.06 ^d^ (0.77–0.93)	119 ± 6 ^bcd^ (113–126)	22.7	1/8% [0–40%]	11/92% [60–100%]
AO90	53.5 ± 4.3 ^bcd^ (48.1–58.8)	0.92 ± 0.05 ^de^ (0.85–0.98)	121 ± 7 ^bcd^ (112–130)	20.7	4/33% [8–67%]	8/67% [33–92%]
AO110a	51.2 ± 3.8 ^bcd^ (46.4–56.0)	1.11 ± 0.07 ^fg^ (1.02–1.20)	122 ± 9 ^cd^ (108–133)	20.5	3/25% [4–59%]	9/75% [41–96%]
AO110b	53.1 ± 2.2 ^bcd^ (50.4–55.9)	1.16 ± 0.03 ^g^ (1.12–1.19)	121 ± 11 ^bcd^ (106–136)	17.1	3/25% [4–59%]	9/75% [41–96%]
AO120a	44.7 ± 6.0 ^ab^ (37.3–52.1)	1.02 ± 0.77 ^efg^ (0.92–1.11)	114 ± 7 ^abc^ (102–123)	22.6	4/33% [8–67%]	8/67% [33–92%]
AO120b	52.1 ± 3.1 ^bcd^ (48.3–55.9)	0.95 ± 0.02 ^de^ (0.93–0.97)	111 ± 5 ^ab^ (104–120)	11.8	3/25% [4–59%]	9/75% [41–96%]
AO125	52.4 ± 2.3 ^bcd^ (49.6–55.3)	1.40 ± 0.14 ^h^ (1.23–1.57)	120 ± 3 ^bcd^ (112–126)	32.1	6/50% [20–80%]	6/50% [20–80%]
SO50a	48.8 ± 3.9 ^abcd^ (43.9–53.7)	0.15 ± 0.05 ^ab^ (0.09–0.20)	117 ± 12 ^bcd^ (106–128)	13.1	4/33% [8–67%]	8/67% [33–92%]
SO50b	56.2 ± 3.8 ^cd^ (51.4–61.1)	0.12 ± 0.01 ^a^ (0.11–0.14)	116 ± 5 ^abc^ (107–124)	25.5	5/42% [14–74%]	7/58% [26–86%]
SO100	52.3 ± 6.0 ^bcd^ (37.3–52.1)	0.11 ± 0.13 ^a^ (0.09–0.12)	121 ± 8 ^bcd^ (110–132)	21.9	5/42% [14–74%]	7/58% [26–86%]
SO100/200	40.3 ± 5.0 ^a^ (34.2–46.5)	0.46 ± 0.06 ^c^ (0.39–0.53)	106 ± 11 ^a^ (89–124)	14.5	4/33% [8–67%]	8/67% [33–92%]
NS100/200	56.7 ± 6.2 ^cd^ (49.1–64.4)	0.14 ± 0.01 ^ab^ (0.13–0.15)	121 ± 7.0 ^bcd^ (110–130)	22.6	4/33% [8–67%]	8/67% [33–92%]
Hydrofluoric acid-etched	57.6 ± 4.1 ^d^ (52.5–62.8)	0.52 ± 0.09 ^c^ (0.41–0.63)	123 ± 6.8 ^d^ (113–131)	19.7	6/50% [20–80%]	6/50% [20–80%]
Polished	57.5 ± 2.8 ^d^ (54.1–61.0)	0.04 ± 0.01 ^a^ (0.03–0.04)	118 ± 6.9 ^bcd^ (111–126)	21.9	3/25% [4–59%]	9/75% [41–96%]

Different superscript lowercase letters indicate significant differences in columns (*p* < 0.05).

## Data Availability

The original contributions presented in the study are included in the article, further inquiries can be directed to the corresponding author.

## References

[1] Peumans M., Valjakova E.B., De Munck J., Mishevska C.B., Van Meerbeek B. (2016). Bonding effectiveness of luting composites to different CAD/CAM materials. J. Adhes. Dent..

[2] Ural Ç., Duran İ., Evmek B., Kavut İ., Cengiz S., Yuzbasioglu E. (2017). Light transmittance and surface roughness of a feldspathic ceramic CAD-CAM material as a function of different surface treatments. BMC Oral Health.

[3] Shi H.Y., Pang R., Yang J., Fan D., Cai H., Jiang H.B., Han J., Lee E.-S., Sun Y. (2022). Overview of several typical ceramic materials for restorative dentistry. BioMed Res. Int..

[4] Moravej-Salehi E., Moravej-Salehi E., Valian A. (2016). Surface topography and bond strengths of feldspathic porcelain prepared using various sandblasting pressures. J. Investig. Clin. Dent..

[5] Azevedo V.L.B., de Castro E.F., Bonvent J.J., de Andrade O.S., Nascimento F.D., Giannini M., Cavalli V. (2021). Surface treatments on CAD/CAM glass-ceramics: Influence on roughness, topography, and bond strength. J. Esthet. Restor. Dent..

[6] Stawarczyk B., Beuer F., Ender A., Roos M., Edelhoff D., Wimmer T. (2013). Influence of cementation and cement type on the fracture load testing methodology of anterior crowns made of different materials. Dent. Mater. J..

[7] Sen N., Us Y.O. (2018). Mechanical and optical properties of monolithic CAD-CAM restorative materials. J. Prosthet. Dent..

[8] Sağlam G., Cengiz S., Köroğlu A., Şahin O., Velioğlu N. (2023). Comparison of the micro-shear bond strength of resin cements to CAD/CAM glass ceramics with various surface treatments. Materials.

[9] Queiroz-Lima G., Strazzi-Sahyon H.B., Maluly-Proni A.T., Fagundes T.C., Briso A.L.F., Assunção W.G., Delben J.A., Santos P.H.D. (2022). Surface characterization of indirect restorative materials submitted to different etching protocols. J. Dent..

[10] Dönmez M.B., Yucel M.T., Kilic I., Okutan Y. (2018). Novel ceramic primer vs. conventional treatment methods: Effects on roughness and bond strength of all-ceramic restorations. Am. J. Dent..

[11] Bagheri H., Hooshmand T., Aghajani F. (2015). Effect of ceramic surface treatments after machine grinding on the biaxial flexural strength of different CAD/CAM dental ceramics. J. Dent..

[12] Addison O., Marquis P.M., Fleming G.J.P. (2007). The impact of modifying alumina air abrasion parameters on the fracture strength of a porcelain laminate restorative material. Dent. Mater..

[13] Donmez M.B., Okutan Y., Yucel M.T. (2020). Effect of prolonged application of single-step self-etching primer and hydrofluoric acid on the surface roughness and shear bond strength of CAD/CAM materials. Eur. J. Oral Sci..

[14] Stawarczyk B., Hristova E., Sener B., Roos M., Edelhoff D., Keul C. (2014). Effect of hydrofluoric acid etching duration on fracture load and surface properties of three CAD/CAM glass-ceramics. Oral Health Dent. Manag..

[15] Özcan M., Allahbeickaraghi A., Dündar M. (2012). Possible hazardous effects of hydrofluoric acid and recommendations for treatment approach: A review. Clin. Oral Investig..

[16] Loomans B., Mine A., Roeters F., Opdam N., De Munck J., Huysmans M., Van Meerbeek B. (2010). Hydrofluoric acid on dentin should be avoided. Dent. Mater..

[17] Saracoglu A., Özcan M., Kumbuloglu O., Turkun M. (2011). Adhesion of resin composite to hydrofluoric acid-exposed enamel and dentin in repair protocols. Oper. Dent..

[18] Abdulla M.A., Hasan R.H. (2022). Shear bond strength of two repair systems to zirconia ceramic by different surface treatments. J. Lasers Med. Sci..

[19] Carrabba M., Vichi A., Louca C., Ferrari M. (2017). Comparison of traditional and simplified methods for repairing CAD/CAM feldspathic ceramics. J. Adv. Prosthodont..

[20] Kamath V., Kamath M., Bhargava A., Shetty T., Rodrigues S.J., Pai U.Y., Saldanha S., Mahesh M., Hegde P., Bajantri P. (2022). An in vitro study on the shear bond strength of feldspathic porcelain to nickel chromium alloy and cobalt chromium alloy after various surface treatments. Int. J. Dent..

[21] Turunç Oğuzman R., Yüzbaşıoğlu E. (2023). Air-polishing powders’ effect on the color of CAD/CAM restorative materials. Appl. Sci..

[22] Aswal G., Nair C. (2015). Effects of various parameters of alumina air abrasion on the mechanical properties of low-fusing feldspathic porcelain laminate material. S. Afr. Dent. J..

[23] Hoffmann M., Stawarczyk B., Günster J., Zocca A. (2024). Influence of additives and binder on the physical properties of dental silicate glass-ceramic feedstock for additive manufacturing. J. Mech. Behav. Biomed. Mater..

[24] Lankes V., Reymus M., Liebermann A., Stawarczyk B. (2023). Bond strength between temporary 3D printable resin and conventional resin composite: Influence of cleaning methods and air-abrasion parameters. Clin. Oral Investig..

[25] (1998). Geometrical Product Specifications (GPS)—Surface Texture: Profile Method—Nominal Characteristics of Contact (Stylus) Instruments.

[26] Wendler M., Belli R., Petschelt A., Mevec D., Harrer W., Lube T., Danzer R., Lohbauer U. (2017). Chairside CAD/CAM materials. Part 2: Flexural strength testing. Dent. Mater..

[27] Coldea A., Fischer J., Swain M.V., Thiel N. (2015). Damage tolerance of indirect restorative materials (including PICN) after simulated bur adjustments. Dent. Mater..

[28] Hoffmann M., Coldea A., Dönmez M.B., Meinen J., Stawarczyk B. (2024). Mechanical properties of high- and low-fusing zirconia veneering ceramics fired on different trays and substrates. Materials.

[29] Hoffmann M., Mayinger F., Stawarczyk B. (2024). Influence of different surface finishing procedures of strength-gradient multilayered zirconia crowns on two-body wear and fracture load: Lithium silicate or leucite glazing versus polishing?. J. Mech. Behav. Biomed. Mater..

[30] (2015). Dentistry-Ceramic Materials.

[31] Valandro L.F., Cadore-Rodrigues A.C., Dapieve K.S., Machry R.V., Pereira G.K.R. (2023). A brief review on fatigue test of ceramic and some related matters in Dentistry. J. Mech. Behav. Biomed. Mater..

